# A Distributed and Secure Self-Sovereign-Based Framework for Systems of Systems

**DOI:** 10.3390/s23177617

**Published:** 2023-09-02

**Authors:** Dhiah el Diehn I. Abou-Tair, Raad Haddad, Ala’ Khalifeh, Sahel Alouneh, Roman Obermaisser

**Affiliations:** 1 School of Electrical Engineering and Information Technology, German Jordanian University, Amman 11180, Jordan; ala.khalifeh@gju.edu.jo (A.K.); sahel.alouneh@gju.edu.jo (S.A.); 2 Cloudyrion GmbH, 40221 Düsseldorf, Germany; r.haddad@cloudyrion.com; 3 College of Engineering, Al Ain University, Abu Dhabi 112612, United Arab Emirates; 4 Faculty of Science and Technology, University of Siegen, 57076 Siegen, Germany; roman.obermaisser@uni-siegen.de

**Keywords:** security, privacy, blockchains, distributed ledger, permission, system of systems

## Abstract

Security and privacy are among the main challenges in the systems of systems. The distributed ledger technology and self-sovereign identity pave the way to empower systems and users’ security and privacy. By utilizing both technologies, this paper proposes a distributed and self-sovereign-based framework for systems of systems to increase the security of such a system and maintain users’ privacy. We conducted an extensive security analysis of the proposed framework using a threat model based on the STRIDE framework, highlighting the mitigation provided by the proposed framework compared to the traditional SoS security. The analysis shows the feasibility of the proposed framework, affirming its capability to establish a secure and privacy-preserving identity management system for systems of systems.

## 1. Introduction

Systems of systems (SoS) aim to achieve functions and services by integrating multiple constituent systems (CSs). Each CS possesses various resources and services that SoS’s users can independently access. These CSs are interconnected, and the SoS coordinates their resources and services to comprehensively understand all available resources and services [1,2,3]. The primary advantage of adopting an SoS is its ability to utilize the resources of individual systems while considering numerous factors, such as the cost, availability, reliability, safety, privacy, and security of resources. This has increased the popularity of SoS applications in various domains, such as healthcare, aerospace and automotive manufacturing, Industry 4.0, defense, and security systems [4,5,6]. For instance, one healthcare system might provide health-related measurements, and another for data analysis and processing, while a third system makes decisions for specific healthcare cases. This enables the relevant healthcare personnel to detect and relay emergency conditions.

However, ensuring the security of SoS is complex due to the dynamic and diverse nature of the SoS architecture, as each CS has its security measures and configurations. These measures, designed to ensure the security of individual systems, may not apply to the dynamic environment of the SoS. Consequently, there is an essential need for a universal security framework for SoS that ensures the security of the individual CSs and the SoS as a whole.

One of the significant concerns of SoS’s security is managing the customers’/users’ identities. Therefore, a robust identity management system (IDMS) is crucial for the overall security of the SoS. In such a system, users within the CSs have digital identities that enable them to interact and access the resources of the CSs. Within the SoS ecosystem, an IDMS manages users’ identities across different CSs networks via rules associated with users’ digital identities and credentials. The distributed topology of the SoS requires a decentralized IDMS, which differs from the majority of centralized IDMSs. Centralized IDMSs often need more scalability, single points of failure, and vulnerability to identity theft attacks, making them unsuitable for a distributed and scalable SoS environment.

In the proposed framework, a decentralized IDMS is realized based on self-sovereign identity (SSI) using digital ledger technology (DLT) [7]. The benefit of using SSI is that it preserves users’ privacy by granting them full control over sharing their data. It also implies that CSs can verify users’ data without storing them, maintaining a stateless system. Users can then access different resources in the SoS CSs without compromising their private information. For instance, a user could verify their age to access a resource within a CS without revealing their actual date of birth.

This paper proposes a secure, distributed, self-sovereign-based framework for SoS. The system architecture serves the needs of the SoS by providing a scalable, secure, privacy-preserving, and decentralized identity management system that maintains users’ privacy and security.

The proposed framework addresses an essential SoS security feature, namely the right to access a service from a security perspective, that is, equipping the SoS with a dynamic access control mechanism. Furthermore, the proposed framework preserves users’ privacy by utilizing self-sovereign identity technology, wherein the user is not required to disclose their private information to access a service. Instead, the user needs to demonstrate that they are entitled to access the service via a verifiable proof.

The proposed framework is implemented using Hyperledger Indy (https://www.hyperledger.org/use/hyperledger-indy (accessed on 1 May 2023)). To demonstrate the feasibility of the proposed framework, a security analysis was conducted to identify and analyze potential threats and risks. Additionally, a threat model based on the STRIDE framework was carried out, highlighting the mitigation provided by the proposed SoS security framework compared to an SoS utilizing traditional centralized security measures.

The rest of this paper is organized as follows. Section 3 summarizes the most relevant papers in the literature. The proposed security framework is presented in Section 3. The system implementation and evaluation are discussed in Section 5. Finally, this paper is concluded in Section 6.

## 2. Security Challenges of Systems of Systems

Systems of systems are large-scale collaborative systems where autonomous constituent systems work together to provide emerging services that exceed the local services of the constituent systems. The CSs can be geographically distributed and belong to different organizations. Significant challenges are the lack of central control and information about the internals of CSs, which prevent the centralized establishment of services. SoS is increasingly important in different domains, such as transportation systems, smart grids, smart production, healthcare, and defense systems. Many of these systems also exhibit non-functional requirements such as stringent temporal constraints and reliability requirements.

Since SoS plays an essential role in critical infrastructure and offers safety-relevant services, the security in SoS must be considered. Firstly, attackers may affect the availability of SoS using denial-of-service attacks. In addition, attackers can interfere in negotiating service contracts and providing services between constituent systems. Therefore, the authenticity of service providers and service users must be ensured during the cooperation of constituent systems. Finally, sensitive information, such as medical records in healthcare applications, can be communicated. Therefore, secure services are required to ensure security and privacy.

In particular, present-day SoS face the following security challenges:

- Confidentiality: Traditional systems encountered significant challenges in maintaining confidentiality. System owners and developers had to encrypt all user-related information and store it securely in inaccessible locations to prevent unauthorized access. Their most significant challenge was if encryption keys were compromised or weak encryption algorithms were used, which put all the information at risk of potential leakage or unauthorized modification. Our proposed approach enhances the confidentiality of user data and access keys by storing them in digital wallets located on the user’s side in a secure, encrypted manner.
- Integrity: Centralized systems were plagued with privilege escalation and data manipulation issues, facilitating numerous malicious activities. Our proposed approach gives users exclusive control over their identities, preventing them from being shared or stored elsewhere. Moreover, data alteration will not affect the process, as it will continually verify the submitted verifiable proof on the DLT. Any detected modification will cause the authentication and authorization processes to fail, thus inhibiting further progression.
- Availability: When centralized systems experience downtime, users cannot authenticate themselves until the issue is resolved. However, the proposed approach, fortified with blockchain technology, makes it considerably more difficult, or even impossible, for attackers to disrupt the service or make it unusable for users, as it would require significant computing power and resources.

The introduced services support security processes for addressing security risks in SoS, such as OASoSIS [8]. The proposed security framework, with its encryption, identity management, and authentication services, represents a mitigation approach for reducing risks to SoS stakeholders.

## 3. Related Work

Systems of systems (SoS) solutions have attracted considerable research interests [9,10,11,12,13,14,15,16,17,18,19,20,21]. A study by Olivero et al. [9] addressed the problem of assessing security properties in SoS. It proposed a Testing Security in the System of Systems (TeSSoS) approach, which included modeling and testing security properties in SoS. TeSSoS adopted the perspective of attackers to identify security flaws and propose the development of new features. The authors aimed to provide an approach for assessing SoS security and continuing its development, paying particular attention to security testing, modeling security features, evaluating human factors relevance, and implementing control policies.

Guariniello and DeLasurentis [10] analyzed the implications of cyber-attacks on SoS. They utilized a modified functional dependency analysis tool to model the tertiary effects of such attacks. Their study primarily focused on risk assessment and did not specifically address the security requirements of the SoS. The authors evaluated the robustness of the SoS in terms of its ability to sustain an acceptable level of operation after a communication disruption has occurred.

In their work [11,12], Trivellato et al. presented a service-oriented security framework that aims to safeguard the information shared between entities within an SoS while also ensuring the preservation of their autonomy and interoperability. To showcase the practical viability of the framework, the authors implemented it within the context of the maritime safety and security domain. By doing so, they demonstrated the applicability of the SoS in this particular domain.

EL Hachem et al. [13] proposed a Model Driven Engineering method called Systems-of-Systems Security (SoSSec). This method was designed to model and analyze secure SoS solutions, particularly in predicting high-impact cascading attacks during the architecture phase. In their study, the authors demonstrated the effectiveness of the proposed method by applying it to a real-life smart building SoS. The case study showed that the SoSSec method successfully identified and analyzed the cascading attacks consisting of multiple individual attacks.

In [14], Nguyen et al. performed a systematic mapping study (SMS) that aims to evaluate the current state of Model-Based Security Engineering (MBSE) for Cyber-Physical Systems (CPSs). The work showed a significant increase in primary studies related to MBSE for CPSs, mainly in the security analysis. However, their work revealed a need for more engineering security solutions for CPSs. Furthermore, the SMS highlighted several critical issues, such as the limited availability of tool support and the challenge of integrating domain-specific languages (DSLs) to secure CPSs effectively.

In [16], Bicaku et al. proposed an automated and continuous standard compliance verification framework based on a set of technically measurable indicators from security standards. This framework enabled the verification of system compliance with various security standards. Several advantages of the framework have been emphasized, such as continuous monitoring, automation capabilities, and extensibility. Furthermore, the authors analyzed several implementation-related challenges, such as the necessity for accurate and up-to-date information regarding the standards. Consequently, this framework underlined the significance of ensuring the compliance of SoS with security standards, presenting it as a more effective and efficient alternative to traditional manual approaches.

Agrawal et al. [17] put forward a security schema for SoS that addresses the dynamic and uncertain nature of the environment. Unlike the traditional approach of static security, their schema incorporated mechanisms that continuously monitored the overall environment and used the collected observations to adjust the security posture dynamically. This recognition of the ever-changing threat landscape distinguished their schema from the static security approaches. The authors hypothesized that adopting such security schemata would enable a systematic analysis of the security of complex systems and provide a quantified assessment of the resilience of the security within an SoS.

Maesa et al. [20] presented a Blockchain-based access control protocol that utilized the resource access policies and rights of public publication on the Blockchain. This approach enabled users to have real-time access to the resources’ pairing information and policies, as well as the authorized personnel to access those resources. By leveraging Blockchain transparency and immutability, the protocol delivered reliable and accessible access control management mechanisms.

Xu et al. [21] introduced the concept of Distributed Ledger-Based Access Control (DL-BAC) specially designed for web applications. The proposed DL-BAC offered a decentralized access control mechanism while ensuring users’ privacy. Furthermore, by utilizing distributed ledger technology, DL-BAC provided a secure and privacy-preserving approach to access control in web applications, thus offering an alternative solution that eliminated the need for a central trusted entity.

In our previous work [15], we proposed a systems-of-systems security framework that utilizes multi-protocol label switching (MPLS). The main objective of the proposed framework was to offer several advantages, including connectivity, reliability, and quality of service. In addition, it included features such as traffic separation and isolation while minimizing management and processing overhead. Furthermore, an advanced security configuration for complex scenarios has been proposed by integrating IPsec and the MPLS, enhancing overall security. However, it is important to mention that our work did not consider the SoS identity management or the associated access control challenges. Additionally, we did not consider other threats, such as denial-of-service attacks, which can impact network services like the domain name system (DNS).

Furthermore, in our other previous research discussed in [18], we proposed a distributed access control system that utilizes Blockchain technology to ensure secure and privacy-preserving management of access to distributed resources. The system was specifically designed to be decentralized and distributed, enhancing its security and resilience against potential attacks.

This work builds on our previous works [18,22] by proposing a new framework for a secure, distributed, self-sovereign-based SoS. The proposed system architecture serves the specific needs of SoS by providing a scalable, secure, privacy-preserving, and decentralized identity management system. The main objective is to protect users’ privacy and security while ensuring the necessary functionality for the SoS.

## 4. The Systems-of-Systems Security Framework

### 4.1. The Proposed Framework

**Figure 1:**
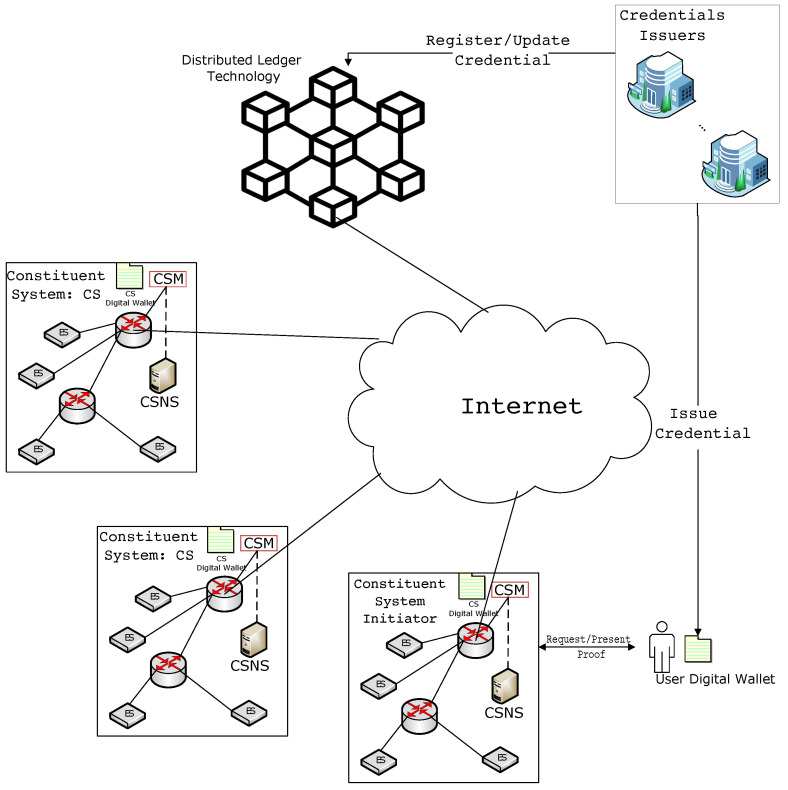
The proposed SoS security framework.

The proposed framework leverages distributed ledger technology to address security and privacy challenges in the context of SoS. The dynamic and distributed nature of SoS necessitates a decentralized security mechanism capable of fulfilling the security and privacy requirements of the SoS environment. For instance, users may access multiple resources distributed across different constituent systems; thus, the serving CSs must verify their identities. Furthermore, the resources may require specific access credentials from the users, who should be able to present access permission without compromising their private information. Scalability is another vital factor in SoS due to its scalable nature, where users can access many available resources and services distributed among several CSs. These requirements are considered in the proposed decentralized self-sovereign-identity-based security framework. Figure 1 depicts the proposed SoS security framework architecture. The framework consists of several connected CSs, which are also connected to a distributed ledger network. Additionally, the framework consists of credential issuers (CIs) and service requesters (users). The role of the credential issuer is to issue digital credentials for users registered inside the distributed ledger network and stored in the individual user’s wallet as its sole owner. The user can use the credentials to create verifiable proof to gain access to SoS resources. For instance, a verifiable proof can be derived from the user’s birth certificate, which shows that the user is above a certain age limit without revealing the actual date of birth. Moreover, the credentials could incorporate SoS resources’ access control information to create a verifiable proof to access the resources. In what follows, the framework’s main components are described.

#### 4.1.1. Distributed Ledger Technology and Blockchain

Distributed ledger technology is an emerging technology for storing data in replicated databases (ledgers or data stores) across multiple sites managed by a distributed server network (nodes). The main advantage of DLT is its decentralized nature for storing, sharing, and synchronizing data across multiple nodes, utilizing a peer-to-peer communication paradigm. Blockchain is one type of DLT that transmits and stores data packages named Blocks. These Blocks are joined together to form an append-only digital chain. For data recording and synchronization across the Blockchain network, Cryptographic and algorithmic methods are used [7].

#### 4.1.2. Self-Sovereign Identity

SSI is a concept that enables users to have complete control over their identities and personal data and enables services to control who can access them without the intervention of a mediator (third party) [23]. This is achieved by storing the users’ identities in digital wallets owned by the users and the services’ access requirements in digital wallets owned by CSs. When users/services try to access a resource or service, they generate a verifiable proof utilizing the credentials stored in their digital wallets in response to a proof request from the verifier. The verifier in the context of the proposed SoS framework is the Broker or the CSM, which will process the response data and check its authenticity, thus allowing or denying access to the requested resources or services.

#### 4.1.3. Credentials’ Issuers

Credential issuers are trusted entities that issue verifiable credentials in response to a user’s credential request. Verifiable credentials include birth certificates, bank accounts, personal identities (e.g., government IDs, passports, and social security credentials), insurance policy certificates, access control information, etc. These verifiable credentials are stored in users’ digital wallets, from which verifiable proofs required by resources are derived. For the proof verification process, CIs will register the credentials on the DLT.

#### 4.1.4. The Digital Wallet

Both users and CSs have digital wallets to store verifiable credentials. In the context of SoS, some resources may require certain credentials. If the user accesses such a resource, they must provide proof of the required credentials, which can be derived from the verifiable credentials stored in their wallet. As for CSs, the digital wallet is needed to store their verifiable credentials, which enable them to identify themselves to other CSs to use their services. The users, bearing responsibility for their digital wallets containing their verifiable credentials, are advised to link one of their biometric attributes, such as a fingerprint, to access their digital wallet. This precautionary measure will mitigate the potential misuse of user credentials in the event of unauthorized access to the digital wallet device.

#### 4.1.5. The Broker

The Broker, referred to as the CS Initiator, is responsible for accepting users’ service requests and contacting CSs to provide service offers that match the requests. The CS Initiator then selects the optimal service offers based on the user’s predefined criteria, such as cost and execution time. Additionally, the CS Initiator plays a vital role in ensuring users’ overall security and privacy by validating the general credentials requested by the CSs. Once the Broker receives the service offers from the CSs, it will ask the user to provide the necessary proof that allows them to access the resources. The Broker will then forward the user proofs to the CSs, verifying them via the DLT. Once the proofs are verified, the CSs will allow the user to access the requested services. In the proposed framework, each CS has a digital wallet, which includes its identity as verifiable credentials issued by the SoS service provider as a CI and used within the SoS network, thus creating a trustworthy communication paradigm between the CSs.

#### 4.1.6. Constituent System Manager

The CSM handles all communication between the CS and the Broker. The CSM ensures that the requested resources or services are available for usage. Furthermore, each CS has specific security requirements to access resources or services. Also, CSM plays a role in the security framework, as it is responsible for verifying the specific proofs provided by the user on the DLT. As each CSM can verify its security requirements using DLT in a decentralization manner, this improves the overall security of the framework.

### 4.2. The Framework Work Flow

**Figure 2:**
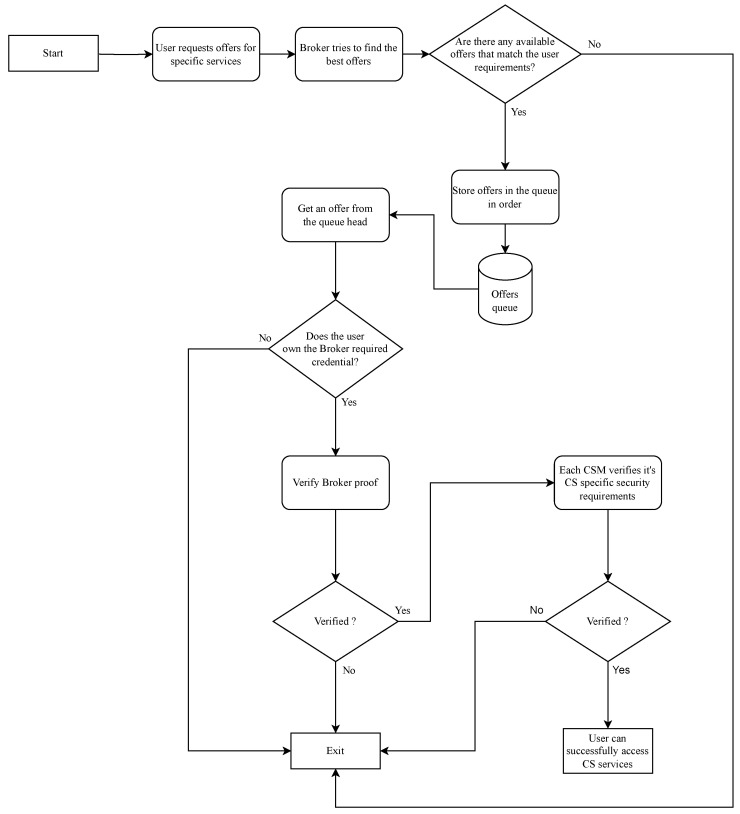
The workflow of the proposed framework.

Figure 2 shows the workflow of the proposed framework which can be summarized as follows:

- Credential issuers issue verifiable credentials that are stored in users’ wallets and registered on the DLT.
- The user will connect with the CS Initiator (Broker) to request the required service from the CSs.
- The Broker will contact the CSs and request offers of services pertaining to the user request, mentioning the execution time and cost of each service. The CSs’ responses will be queued according to the optimization criterion set by the application under consideration. For example, the offers with the least computational cost will be queued in ascending order according to the execution time. The Broker will then select the best offer that matches the request’s requirement and constraints by solving a constraints optimization problem, where the main objective function may vary depending on the application requirements. Further details about the selection and optimization of offers can be found in our previous work [22].
- The Broker will provide the user with the queued offers and their associated privacy and security requirements. The user will then be able to evaluate the offers’ security and privacy requirements to best suit their security and privacy needs.
- The Broker will request the user to provide a verifiable proof that indicates they possess the necessary access credentials for the offered services. The Broker will verify the user proof on behalf of the requested CS service provider. Additionally, the Broker may ask the user to provide a verifiable proof, which will be sent directly to the CS service provider for verification. These two types of proofs are distinguished in the implementation Section 5.1 as general and specific proofs, respectively.
- The verifiable proofs will be verified by the Broker or CSs using the DLT.

## 5. System Implementation and Evaluation

### 5.1. Implementation

The testbed was implemented on the Ubuntu operating system, and the test machine was equipped with a dual-core processor and 4 gigabytes of RAM. Through Docker, we established a dedicated network solely for this experiment, ensuring effective network isolation and resource segregation implementation. The proposed framework was implemented using Hyperledger Indy, an open-source project focusing on distributed DLT. Hyperledger Indy’s DLT served as the foundation for adding the required nodes and entities to the framework. This allowed for the creation and assignment of credentials to users, which could then be stored in their wallets. Additionally, the distributed ledger was utilized for authenticating identities via the Broker and CS managers, as described in Section 4.1. The implementation leveraged the Indy SDK for Python, which provided the necessary functions for interacting with the distributed ledger.

The implementation comprises several key components. Firstly, there is the Broker, which assumes the responsibility of initiating communication between the CSs and the users. This function ensures mutual trust and conducts the necessary verification process. Additionally, as specified by the user, the Broker retrieves all available services or resources from different CSs. Furthermore, the Broker selects the best offers based on multiple factors, ensuring an optimal offer for each requested asset.

In this implementation, the various services offered by different CSs were equipped with access control requirements. This means that only users who can provide the necessary proof of having the access credentials can access the requested services. The Broker in this implementation has additional functionality to gather the security requirements (access control requirements) for each desired service, along with the optimal offer. The Broker also maintains a risk assessment of sharing each user’s data to facilitate its operations and help users choose a service from a CS that requires less user data and provides the best security options. This prioritizes the user’s privacy and security, as outlined in the proposed framework workflow depicted in Figure 2.

However, it is important to note that CSs have the ability to offer services to users and include all the necessary service requirements. While a particular CS may not provide all the services, it may still offer the best option for a specific service if available. All the requirements are stored within each CS’s Metadata and provided to the Broker whenever a user requests a specific service. Each CS has a dedicated CS manager who handles all communication between the CS and the Broker. The CS manager ensures that the requested service is available for usage, and if it is not, an offer that is not ready will not be presented.

Additionally, the users’ identities and communication data are kept secure via encrypted and secured communication channels. To achieve this, users have a credential containing their personal information. This credential can be used to generate verifiable proof when requested. Following the principles of SSI, it is the user’s responsibility to provide this verifiable proof, also known as a claim, to the verifier, which, in this case, is the Broker. The Broker then authenticates the necessary information with the CS managers.

### 5.2. Use Case and Evaluation of Health Care Services for SoS

**Figure 3:**
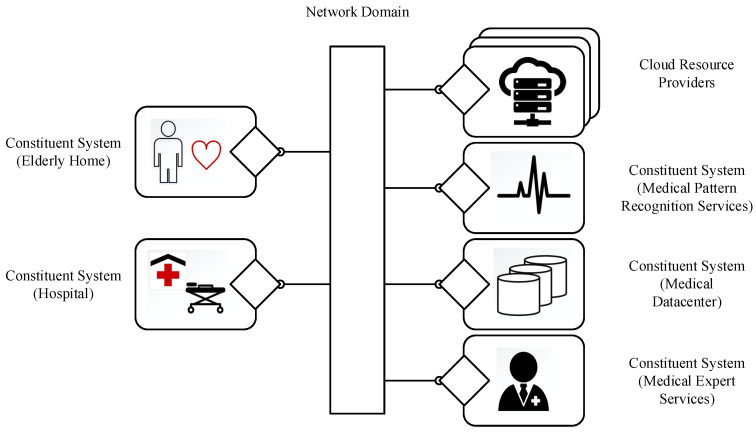
SoS for healthcare services use case.

Figure 3 illustrates a practical use case in healthcare. It depicts a scenario where an elderly individual with a heart condition needs to be monitored for potential heart attacks. A pattern recognition service is used to identify heart attack symptoms; if an emergency occurs, the relevant hospital should be notified. This use case involves finding a suitable pattern recognition service for monitoring heartbeats, utilizing an expert system to analyze the patient’s medical history, and discovering an emergency service provided by a nearby hospital. Establishing a reliable SoS-application will provide the most appropriate services for the desired application, specifically for the medical monitoring of the elderly person. In this use case, each CS should have a CSM, which is the primary processing component responsible for service discovery, inter-networking with other CSs using routers, admission control, and scheduling.

This use case presents several significant challenges for security, including:

- Confidentialityof information is necessary for protecting the privacy of the elderly. This includes safeguarding behavioral patterns like the locations and activities of elderly individuals. Furthermore, the SoS must ensure that medical information is not disclosed.
- Availability is crucial to ensure the proper delivery of safety-related services even in the face of denial-of-service attacks. Any disruption in recognizing health issues and emergency response would pose a medical risk to elderly individuals. For instance, if a denial-of-service attack causes delays in the pattern recognition service’s response time, the entire healthcare system may become unresponsive and fail to identify and address medical emergencies promptly.
- Authenticity is necessary to prevent financial losses resulting from illegitimate interactions that impose costs on elderly individuals or insurance companies. For instance, attackers may initiate unnecessary cloud services, leading to unnecessary expenses. Similarly, authenticity is crucial in blocking illegitimate service providers who offer unreliable services in the health monitoring context. An example would be a low-quality pattern recognition service that compromises the overall accuracy of the health monitoring system.

Addressing these security challenges is critical to ensure the successful implementation and functioning of the healthcare SoS.

This use case has been evaluated using the proposed framework implementation to prove its feasibility and scalability. To this end, the medial use case scenario has been applied to the aforementioned developed testbed, where a patient requires thirty different services from healthcare service providers. In the conducted simulation, the patient’s request was distributed among different CSs according to the services’ availability and compliance with the user requirements in a secure and privacy-preserving manner. To evaluate this, the patient services’ request was assessed by considering one CS providing all requested services, then two, three, and up to thirty CSs providing the requested services in a distributed manner.

When the patient request was initiated, the Broker offered the optimal offers with its security and privacy requirements. On one hand, the Broker verified the patient’s general proof needed to access the SoS services. On the other hand, each CSM verified the specific proof provided by the patient to access the specific CS services.

**Figure 4:**
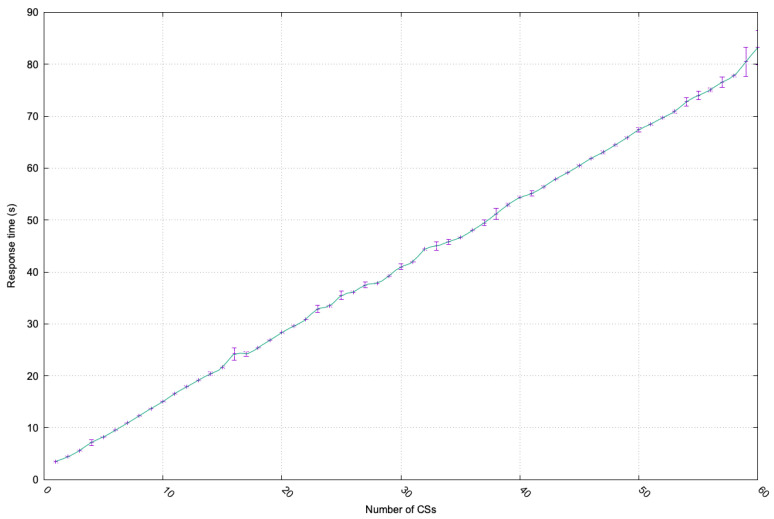
Performance evaluation.

Figure 4 depicts the response time versus the number of CSs used to provide the thirty requested services by the patient. Each experiment was repeated five times to show the results’ variability, which were plotted using an error bar representation. It is observed that the response time increases linearly with the number of used CSs, which verifies the system scalability with the increasing number of CSs. The response time includes the delay incurred in verifying the general and specific proofs via the Broker and the CSMs, respectively, which is time-consuming since it involves accessing the distributed ledger technology network.

**Figure 5:**
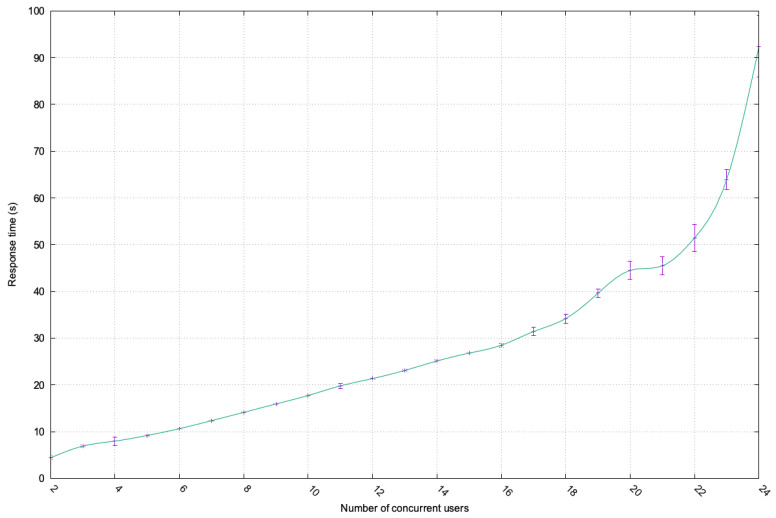
Incremental load testing.

Figure 5 illustrates the response time when the system was overloaded using concurrent users’ service requests. The number of users is increased by one, starting from 2 to 24 concurrent users, who requested the same services in parallel. This demonstrated the proposed framework implementation’s ability to handle multiple users’ service requests in parallel while verifying the services’ security requirements and the users’ authorization to access them via the DLT. It was observed that the response time increased linearly as the number of users increased, which verified the system’s scalability with increasing the number of concurrent users. However, as shown in the figure, when there were 24 concurrent active users, the proposed system reached its saturation point, with an exponential increase in the response time.

### 5.3. Security Analysis

This section conducted a security analysis to investigate the innovative security mechanisms applied within the proposed SoS security framework. The security analysis demonstrated how the proposed framework enhances the SoS environment with robust security features and controls designed to ensure that the authentication and authorization processes of the constituent systems are conducted in a manner that supports both user and system security and privacy. The proposed framework carefully checks and validates the credentials, ensuring that the processes occur securely and privately.

The authorization and authentication processes were historically centralized within the same infrastructure or underlying systems. Over time, organizations and system administrators transitioned to using a dedicated service to exclusively manage the authentication and authorization processes. While this solution represented an improvement, it retained a centralized architecture, storing all user-related data and permissions in one place, making these systems highly attractive targets for attackers. By launching targeted attacks, attackers could carry out various malicious activities, potentially leading to the leakage of sensitive users’ and systems’ data or even discovering vulnerabilities to bypass these mechanisms, impersonate users, escalate privileges, or act maliciously on behalf of other users.

This paper proposes a new methodology for user and system authentication and authorization. It involves the main components that work together to improve the overall security of the SoS to address its dynamic nature. Furthermore, to provide security and protection for the users’ data, the credentials and the communication channels have been identified as essential sources of threats that must be carefully considered.

- Credentials are issued by trusted entities and assigned to the user, and they are securely and exclusively stored on the user’s side in a digital wallet. Digital wallets should use robust encryption algorithms to prevent the use of credentials by unauthorized users in the event of wallet theft or attack. Having the credentials stored on the users’ side will significantly challenge the possible attacker who attacks the SoS infrastructure since the systems don’t include any users’ data. Additionally, during the authentication process, users do not reveal sensitive information such as usernames, passwords, or secret keys; instead, they supply encrypted, verifiable proof. This verifiable proof is generated once and invalidated upon the completion of the verification procedure.
- Communication Channels among the components of the proposed SoS security framework play a significant role in maintaining security and privacy. These channels must be secured and encrypted at all times of communication, which can be accomplished using various methods. The proposed SoS security framework makes use of SSL/TLS to ensure data encryption during data transmission. Given that most communications are managed via APIs, the SoS security framework applies and implements the API security controls across all the endpoints and infrastructure per the OWASP API Top 10 security guidelines (https://owasp.org/API-Security/editions/2023/en/0x11-t10/, accessed on 1 May 2023).

The emphasis on the security considerations in the proposed SoS security framework involves adhering to blockchain security best practices and consistently following guidelines for protecting such infrastructure from various factors, such as human errors, natural disasters, or any other potential impacts. Additionally, we employ APIs in our module and implementation, as is often the case in real-world scenarios. Therefore, securing and hardening APIs is necessary, from receiving requests to returning responses, and communication channels should always be encrypted using state-of-the-art encryption methodologies and technologies.

### 5.4. Threat Model

**Figure 6:**
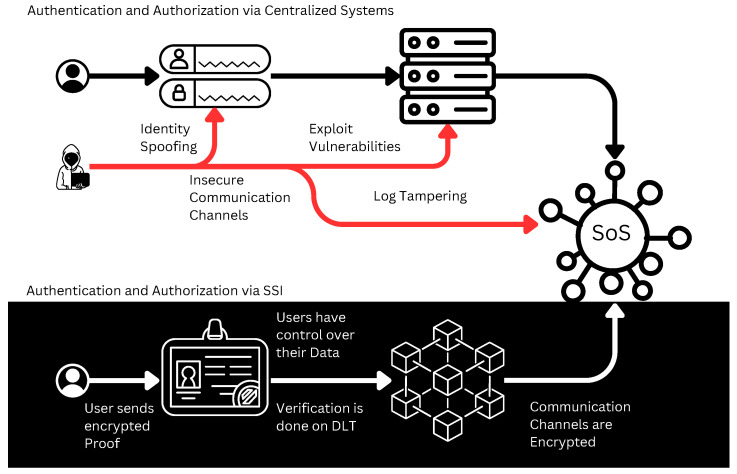
The SoS threat model.

A threat model was conducted for SoS utilizing centralized authentication methods and demonstrated how the proposed SoS security framework presented in this paper assists in mitigating the identified threats as as illustrated in Figure 6 and described in Table 1. The STRIDE Framework was used to identify threats and assess their impacts across the six categories of Spoofing, Tampering, Repudiation, Information Disclosure, Denial of Service (DoS), and Privilege Elevation.

### 5.5. Framework Practicality and Industry Adoption

The proposed framework is practical and can be deployed using current technologies, as it utilizes existing technologies recently used in many applications, such as self-sovereign identity and digital ledger technology. Furthermore, the proposed framework was implemented using Hyperledger Indy, verifying its implementation feasibility. However, integrating the framework into real-world systems of systems, such as automobiles, autonomous ships, manufacturing facilities, energy grids, and medical device networks, poses significant challenges due to the lack of up-to-date communication infrastructure and the absence of an e-government structure and associated legislation. Despite these challenges, many countries are improving and enhancing their infrastructure, which can be seen in the wide adoption of advanced wired and wireless infrastructure, such as fiber optics, fourth and fifth-generation wireless infrastructure, and the deployment of cloud-based networks. This will pave the way for adopting the proposed framework. Moreover, countries are moving toward leveraging their governmental services with an e-government infrastructure and services paradigm.

## 6. Conclusions

In conclusion, the proposed framework provides a secure and scalable solution for managing the identity of users within a SoS environment. By utilizing SSI and DLT, the framework ensures the privacy and control of users’ data while enabling secure interactions between different CSs. Implementing the framework using Hyperledger Indy showcases its feasibility and practicality in real-world scenarios. The security analysis highlights the framework’s ability to address essential security challenges based on the STRIDE framework. By addressing these challenges, the proposed framework enhances the overall security and functionality of SoS. Furthermore, the decentralized and distributed framework provides resilience against centralized attacks and scalability for future expansions. Overall, the framework offers a promising solution to the security concerns in SoS environments and opens up opportunities for broader adoption in other domains. In a future work, we will explore the possibility of adopting and implementing the proposed framework in a real healthcare system and utilize a cloud-based environment with increased computational capabilities, which, in turn, can serve a higher number of concurrent users.

## Figures and Tables

**Table 1:** SoS threat model based on STRIDE framework.

Threat Category	Identified Threats	Mitigations Using the Proposed SSI-Based Framework
Spoofing Identity	Weak encryption algorithms or lack of encryption can increase risk of attack. Man-in-the-Middle (MiTM) attacks can be used to impersonate another user’s identity.	User-related information is transmitted in an encrypted state. Verifiable proof is invalidated once verified by DLT.
Tampering	Session data tampering can be exploited by malicious actors to impersonate other users, compromising system security.	Session tampering infeasible in SSI. Validators can detect false proofs.
Repudiation	Alteration or deletion of user activities within the SoS is possible if security misconfigurations or other types of vulnerabilities occur. Security flaws or failure to adhere to security best practices for the utilized logging and monitoring solutions may lead to the modification of user or system logs.	DLT allows for the creation of an immutable log of identity-related activities. Recording and verifying transactions and interactions can involve multiple parties, generating a traceable record. Establishing a verifiable sequence of events is essential when there are alterations, deletions, or claims of denial.
Information Disclosure	Misconfiguration or improper implementation of centralized authentication and authorization systems can lead to data leakage. Personally Identifiable Information (PII) or clear-text access keys can be exposed. Misuse of this information can compromise user data and the SoS. Administrative access granted can pose a risk to the SoS.	Credentials are securely stored in a digital wallet on the user’s side, employing robust encryption algorithms. Users generate a single proof from the verified credential to authenticate themselves to the SoS. No user data stored on SoS. SSI avoids centralized storage and ensures encryption.
Denial of Service (DoS)	Centralized authentication and authorization systems are vulnerable to DoS attacks. DoS attacks can make these services unavailable to users, particularly when attackers specifically target DNS systems to disrupt their availability. This prevents users from accessing the services under the SoS.	DLTs are resilient to DoS attacks due to their decentralization nature. Transactions are stored across multiple nodes, making it difficult to target and avoid a single point of failure.
Elevation of Privilege	Exploitation of security vulnerabilities to gain unauthorized access. Consumption of resources without legitimate access.	Attempts to change or escalate privileges with false information are prevented. Users have control over what information they share with the SoS. Requests for excessive permissions or access information are monitored and can be rejected.
